# Severe asthma database in Hungary, initial steps

**DOI:** 10.1186/2045-7022-3-S1-P33

**Published:** 2013-05-03

**Authors:** Zsuzsanna Csoma, Balazs Antus, Imre Barta, Csaba Szalai, Agnes Felne Semsei, Iren Herjavecz

**Affiliations:** 1 National Institute for TB and Pulmonology, Asthma Outpatient Clinic, Hungary; 2 National Institute for TB and Pulmonology, Pathophysiology, Hungary; 3 Semmelweis University, Faculty of Genetics, Cell and Immunobiology, Hungary

## Background

Patients with severe asthma represent a significant unmet clinical need, however the evidence base for the management of these patients is small. Published literature on prevalence of severe asthma is quite heterogeneous and we do not have accurate data in Hungary. In 2010 we started to build up a severe asthma database involving patients who were treated in the pulmonary care network in Hungary and met the ATS criteria for severe asthma. The aims were to determine severe asthma prevalence and to define and characterize clinical phenotypes further.

## Methods

The survey was carried out by using a special severe asthma questionnaire regarding information of sex, age, disease onset and duration, lung function, atopy, smoking habits, systemic steroid claim, exacerbations. To date 454 patients were recruited. 354 of them were registered by the pulmonary clinics over the country (group 1) while the remainders (n=100) were registered by the asthma outpatient clinic of our institute (group 2). The latter group was operated as a reference group to verify the reliability of questionnaire data received from other centers. Identification of severe asthma phenotypes was started in patients of group 2, and it is based on more detailed clinical features and determination of pattern of airway inflammation using induced sputum, exhaled breath condensate sample analysis and measurement of exhaled NO.

## Results

There were no differences between the two groups in gender distribution, prevalence of atopy, systemic steroid dependency and the mean value of personal best FEV1. On the other hand the mean age of the group 1 was significantly higher, with no difference in asthma duration between the two groups.

**Table 1:** 

	Group1	Group2	p value
Age (year)	58.3±12.9	51.8±13.3	<0.001
Asthma duration (years)	21.9±11.7	24.5±14.6	>0.05
Best FEV1 (% pred)	66.9±20.8	70.1±17.5	>0.05
Gender (male/female, %)	39/61	35/65	>0.05
Atopy (yes/no, %)	54/46	52/48	>0.05
Systemic steroid dependeny (%)	33/67	28/72	>0.05

Considering that the significant indicators of the two groups proved to be quite homogeneous, the combined clinical data of 454 patients were compared to the data published in the literature (ENFUMOSA, SARP, TENOR studies) and no relevant differences were found. Phenotypes of severe asthma with early onset atopic disease, severe asthma with salicylat intolerance and systemic steroid dependency and severe asthma induced by airway infection and characterized by mixed eosinophilic-neutrophilic airway inflammation and systemic steroid dependency were found.

## Conclusion

Data of patients registered in our severe asthma database are similar to the international data, but a phenotype of severe asthma induced by airway infection was detected.

